# Identification and Characterization of Three Epithiospecifier Protein Isoforms in *Brassica oleracea*

**DOI:** 10.3389/fpls.2019.01552

**Published:** 2019-12-19

**Authors:** Katja Witzel, Marua Abu Risha, Philip Albers, Frederik Börnke, Franziska S. Hanschen

**Affiliations:** ^1^Leibniz Institute of Vegetable and Ornamental Crops, Großbeeren, Germany; ^2^Institute of Biochemistry and Biology, University of Potsdam, Potsdam, Germany

**Keywords:** epithionitrile, expression profile, functional complementation, glucosinolate hydrolysis, nitrile, specifier proteins, tissue specificity

## Abstract

Glucosinolates present in *Brassicaceae* play a major role in herbivory defense. Upon tissue disruption, glucosinolates come into contact with myrosinase, which initiates their breakdown to biologically active compounds. Among these, the formation of epithionitriles is triggered by the presence of epithiospecifier protein (ESP) and a terminal double bond in the glucosinolate side chain. One *ESP* gene is characterized in the model plant *Arabidopsis thaliana* (AtESP; At1g54040.2). However, *Brassica* species underwent genome triplication since their divergence from the *Arabidopsis* lineage. This indicates the presence of multiple ESP isoforms in *Brassica* crops that are currently poorly characterized. We identified three *B. oleracea* ESPs, specifically *BoESP1* (LOC106296341), *BoESP2* (LOC106306810), and *BoESP3* (LOC106325105) based on *in silico* genome analysis. Transcript and protein abundance were assessed in shoots and roots of four *B. oleracea* vegetables, namely broccoli, kohlrabi, white, and red cabbage, because these genotypes showed a differential pattern for the formation of glucosinolate hydrolysis products as well for their ESP activity. BoESP1 and BoESP2 were expressed mainly in shoots, while BoESP3 was abundant in roots. Biochemical characterization of heterologous expressed BoESP isoforms revealed different substrate specificities towards seven glucosinolates: all isoforms showed epithiospecifier activity on alkenyl glucosinolates, but not on non-alkenyl glucosinolates. The pH-value differently affected BoESP activity: while BoESP1 and BoESP2 activities were optimal at pH 6-7, BoESP3 activity remained relatively stable from pH 4 to 7. In order test their potential for the *in vivo* modification of glucosinolate breakdown, the three isoforms were expressed in *A. thaliana* Hi-0, which lacks AtESP expression, and analyzed for the effect on their respective hydrolysis products. The BoESPs altered the hydrolysis of allyl glucosinolate in the *A. thaliana* transformants to release 1-cyano-2,3-epithiopropane and reduced formation of the corresponding 3-butenenitrile and allyl isothiocyanate. Plants expressing BoESP2 showed the highest percentage of released epithionitriles. Given these results, we propose a model for isoform-specific roles of *B. oleracea* ESPs in glucosinolate breakdown.

## Introduction

In the order Brassicales, glucosinolate (GLS) hydrolysis products play a vital role in plant defense, but are also well recognized for their health beneficial effects exerted by the consumption of *Brassicaceae* vegetables. The enzyme myrosinase initiates the breakdown of the sulfur-containing compounds when cells are disrupted and compartmentation is destroyed, for example by herbivore feeding (Wittstock and Burow, 2010; Hanschen et al., 2014). While releasing glucose, a thiohydroximate-*O*-sulfate is formed, which can spontaneously degrade by a Lossen-like rearrangement to an isothiocyanate (ITC) or a nitrile (Rossiter et al., 2007). ITCs are the pungent principle found in mustard, wasabi, and radish (Terada et al., 2015), among others. Moreover, ITCs have antimicrobial, anti-inflammatory, and most frequently investigated, anticarcinogenic properties (Traka and Mithen, 2009; Veeranki et al., 2015). Several *Brassica* vegetables mainly release nitriles and epithionitriles (ETNs) upon GLS hydrolysis (Matusheski et al., 2006; Hanschen and Schreiner, 2017; Klopsch et al., 2017). This is due to the presence of specifier proteins that interact during the degradation of the GLS aglucon (Wittstock and Burow, 2010; Backenköhler et al., 2018). Nitrile specifier proteins (NSPs) were previously identified in *Arabidopsis thaliana* as the evolutionary oldest specifier proteins. Their presence leads to an increased formation of nitriles (Kissen and Bones, 2009; Kuchernig et al., 2012). Occurrence and activity of the epithiospecifier protein (ESP) leads to the generation of ETNs from alkenyl-GLS aglucons as well as nitriles from non-alkenyl-GLS-aglucons (Burow et al., 2006; Matusheski et al., 2006). Many *Brassica* species release nitriles and ETNs upon GLS hydrolysis, among them *B. oleracea* and also *B. campestris, B. carinata,* and *B. rapa* (Macleod and Rossiter, 1985; Matusheski et al., 2004; Hanschen and Schreiner, 2017; Klopsch et al., 2017; Klopsch et al., 2018; Hanschen et al., 2019). So far, ESPs were characterized in *A. thaliana* (Lambrix et al., 2001; De Torres Zabala et al., 2005; Hanschen et al., 2018b) and in *Brassica* species, such as broccoli (*B. oleracea* var. *italica*) (Matusheski et al., 2006) and *B. napus* (Bernardi et al., 2000; Foo et al., 2000). The thiocyanate forming protein (TFP) has evolved from ESP and was reported in *Lepidium sativum*, *Thlaspi arvense,* and *Alliaria petiolate*, where it catalyzes the formation of thiocyanates and ETNs from selected GLS, and nitriles from other GLS (Kuchernig et al., 2011; Kuchernig et al., 2012). A recent study shows that the enlargement in the 3L2 loop of TaTFP is associated with a higher flexibility compared to ESP which enables an alternative loop conformation (alternative to the loop conformation leading to ETN formation due to C-S lyase activity) being the prerequisite for an additional activity as C-C-lyase leading to thiocyanate formation (Eisenschmidt-Bönn et al., 2019).

Tookey was the first to isolate an ESP-rich fraction in 1973 from *Crambe abyssinica* and to show that ESP activity depends on the availability of Fe^2+^ (Tookey, 1973). Fe^2+^ most likely is bound to ESP by the amino acids E260, D264, and H268 (Brandt et al., 2014; Backenköhler et al., 2018). Concerning ESP-catalyzed ETN formation, it is known that the sulfur from its thiirane ring originates from the thioglucosidic sulfur of the GLS (Brocker and Benn, 1983). Thus, Fe^2+^ most likely enables the intramolecular transfer and insertion of the sulfur into the terminal double bond to form the thiirane ring (Brocker and Benn, 1983; Foo et al., 2000; Backenköhler et al., 2018).

Vegetables belonging to *B. oleracea* species (such as broccoli, kohlrabi, Brussels sprouts, white, red, or savoy cabbages) are of high importance with regard to human consumption (FAOSTAT, 2018). As ESP activity reduces formation of health-promoting ITCs (Matusheski and Jeffery, 2001; Matusheski et al., 2006), knowledge on function of specifier proteins in vegetables is essential. Until now, one *ESP* was cloned form *B. oleracea*, expressed in *Escherichia coli* and the recombinant protein was characterized for its role in sulforaphane [4-(methylsulfinyl)butyl isothiocyanate] and sulforaphane nitrile [5-(methylsulfinyl)pentanenitrile] formation (Matusheski et al., 2006). However, it can be assumed that genome triplication of *B. oleracea* (*n* = 9) (Cheng et al., 2014; Liu et al., 2014) resulted in the presence and activity of multiple ESP isoforms with distinct expression patterns as shown for aquaporins (Diehn et al., 2015) and flavonoid biosynthesis genes (Qu et al., 2016).

Here we report the identification of three ESPs from *B. oleracea*. The transcript and protein abundance was investigated in shoots and roots of four *B. oleracea* genotypes, namely broccoli, kohlrabi, white, and red cabbage. Further, heterologous expressed BoESPs were characterized for their substrate specificity towards specific GLS. Finally, their potential for the *in vivo* modification of GLS hydrolysis was tested and the three isoforms were expressed in *A. thaliana* Hi-0, which has very low intrinsic ESP activity.

## Materials and Methods

### Chemicals

Benzonitrile (≥99.9%), 3-butenenitrile (Allyl-CN, ≥98%), 3-(methylsulfanyl)propyl ITC (3MTP-ITC; ≥98%), d/l-dithiothreitol, FeSO_4_(H_2_O)_7_ (≥ 99%), myrosinase (thioglucosidase from *Sinapis alba* seeds; ≥100 units/g), CH_3_COONa(H_2_O)_3_ (≥ 99%), allyl ITC (Allyl-ITC, ≥99%), Coomassie Brilliant Blue R staining solution, Gamborg’s vitamin solution, isopropyl-β-d-thiogalactopyranoside (IPTG), kanamycin sulfate, Murashige and Skoog medium, 4-pentenenitrile (3But-CN, ≥97%), 3-phenylpropanenitrile (2PE-CN) ≥99%), and 2-phenylethyl isothiocyanate (2PE-ITC, ≥99%) were purchased from Sigma-Aldrich Chemie GmbH (Steinheim, Germany). 3-Indoleacetonitrile (IAN) (≥98%) was acquired from Acros Organics (Fischer Scientific GmbH, Schwerte, Germany). 3-Butenyl ITC (3But-ITC, ≥95%) was obtained from TCI Deutschland GmbH (Eschborn, Germany). 3-(Methylsulfinyl)propyl ITC (3MSOP-ITC) and 4-(methylsulfanyl)butyl ITC (4MTB-ITC, ≥98%) were purchased from Santa Cruz Biotechnology (Heidelberg, Germany). 4-(Methylsulfinyl)butyl ITC (4MSOB-ITC) was bought from Enzo Life Sciences GmbH (Lörrach, Germany). (*R*)-5-Vinyloxazolidine-2-thione (*R*-OZT) was purchased from Biosynth AG (Staad, Switzerland). Acetic acid (≥99.5%), 4-hydroxybenzyl GLS (≥99%), methylene chloride (GC Ultra grade), allyl GLS · H_2_O (ROTICHROM^®^ CHR, and vitamin C [l-(+)-ascorbic acid, ≥99%] were obtained from Carl Roth GmbH (Karlsruhe, Germany). 1-Cyano-2,3-epithiopropane [CETP, ≥97.6% (by GC-MS)] was purchased from Taros Chemicals GmbH Co. KG (Dortmund, Germany). 1-Cyano-3,4-epithiobutane (CETB, ≥99%) was purchased from ASCA GmbH Angewandte Synthesechemie Adlershof (Berlin, Germany). Methanol (≥99.95%), acetonitrile (LC-MS grade), and arylsulfatase were purchased from Th. Geyer GmbH & Co. KG (Renningen, Germany). NaSO_4_ (≥99%) was purchased from VWR International GmbH (Darmstadt, Germany). 3-Butenyl (3But), (*R*)-2-hydroxy-3-butenyl- (2OH3But), 4-(methylsulfanyl)butyl- (4MTB), 3-(methylsulfinyl)propyl- (3MSOP), 4-(methylsulfinyl)butyl- (4MSOB), and 2-phenylethyl-GLS (2PE) were purchased from Phytolab GmbH & Co. KG (Vestenbergsgreuth, Germany).

All solvents were of LC-MS or GC-MS grade, ultrapure water was used for all experiments.

### Plant Material

Seeds of white cabbage *B. oleracea* var. *capitata* f. *alba* cv. Jetma (Rijk Zwaan Welver GmbH, Welver, Germany), kohlrabi *B. oleracea* var. *gongylodes* cv. Kolibri, and red cabbage *B. oleracea* var. *capitata* f. *rubra* cv. Integro (both Volmary GmbH, Münster, Germany), and broccoli *B. oleracea* var. *italica* cv. Ironman (Seminis Vegetable Seeds Deutschland GmbH, Neustadt am Rübenberge, Germany) all being F1 hybrids, were germinated on perlite. Sprouts were grown for 8 days in a climate chamber at controlled light (15 h photoperiod, 500 µmol·m^-2^·s^-1^) and temperature regime (day 22°C/night 18°C) as well as 70% humidity. Water was given as needed.

At harvest, sprouts were separated into root and shoot (tissue in between was discarded). Aliquots for RNA (100 mg) and for protein extraction (500 mg) were frozen in liquid nitrogen and stored at -80°C until further sample preparation. For analysis of GLS, an aliquot was weighed and frozen immediately in liquid nitrogen and lyophilized subsequently. Determination of GLS hydrolysis products was performed on 250 mg of fresh plant material, mixed with 250 mg of water and homogenized using a mixer mill at 30 Hz as described earlier (Hanschen and Schreiner, 2017). For determination of ESP activity of *B. oleracea* tissues, 250 mg of tissue was mixed 1:1 with water and ESP-rich plant extracts were prepared as described previously (Hanschen et al., 2018a). Samples from three (protein profiling, GLS, GLS hydrolysis products, ESP activity) or four (transcript profiling) independent experiments were analyzed.

*A. thaliana* seeds of T2 transgenic lines were surface sterilized and germinated on half-strength Murashige Skoog medium (pH 5.8), supplemented with 1.5% sucrose and 50 µg/ml kanamycin, the latter except for Hi-0 wildtype plants. Two weeks after germination, plants were transferred to pots filled with sand and watered with nutrient solution (Gibeaut et al., 1997). Plants were grown for 5 weeks under short-day conditions (8 h light/16 h dark, 22°C, 40–60% humidity). At harvest, roots were carefully removed from pots, blotted dry and samples of rosette leaves and roots were taken and analyzed for GLS breakdown products: 5–55 mg of root and 60–250 mg of shoot tissue were exactly weighed, mixed with water 1: 1, homogenized as described above and analyzed. Three independent experiments were performed to test BoESP activity in Hi-0, with 3–4 replicates consisting of 1–2 plants.

### Analysis of Glucosinolates

To determine the profiles and concentrations of GLS in root and shoot tissue of *B. oleracea*, lyophilized powder was extracted and GLS were analyzed in their desulfo-form (Hanschen and Schreiner, 2017).

### Determination of Glucosinolate Hydrolysis Products in Roots and Shoots

The extraction and quantification of the enzymatically formed GLS hydrolysis products in homogenized plant material or the BoESP activity assay *via* GC-MS was performed as described earlier (Hanschen and Schreiner, 2017) with small modifications: The transfer line was set to 270°C and He flow was 1 ml min^-1^ in the ESP-assay experiments and GLS hydrolysis profile screenings of *B. oleracea*. He flow was 1.8 ml min^-1^ for analyzing GLS hydrolysis products in *A. thaliana*.

### Database Search and Gene Sequence Analyses

The *A. thaliana* ESP nucleotide sequence (At1g54040.2) was used as query to search the *B. oleracea* genome data set at NCBI using BLASTn (https://blast.ncbi.nlm.nih.gov/Blast.cgi). Primers were designed using NCBI Primer-BLAST tool (https://www.ncbi.nlm.nih.gov/tools/primer-blast/). Multiple sequence alignments and assessment of sequence identities were done with MegAlign (DNASTAR, United States) and T-Coffee program (Di Tommaso et al., 2011).

### cDNA Synthesis and qPCR Analysis

RNA was isolated from 100 mg root or shoot material using RNeasy Plant Mini Kit (QIAGEN, Hilden, Germany), according to the manufacturer’s specifications, including DNase treatment. Briefly, cDNA was synthesized from 2 µg total RNA with iScript™ cDNA Synthesis Kit (Bio-Rad Laboratories GmbH, Munich, Germany), according to the manufacturer’s protocol. Transcript abundance of *BoESP*s was estimated by qPCR based on primer pairs detailed in **Table S1**. Two primer pairs were selected for *B. oleracea* reference genes based on geNORM (Vandesompele et al., 2002) analysis of expression stability and previous evaluation (Brulle et al., 2014). The two reference genes chosen were *BoSAND1* (GenBank accession no. KF218596) and *BoTUB6* (GenBank accession no. KF218597). The PCR program comprised an initial denaturation (95°C/3 min) followed by 40 cycles of 95°C/10 s, 54°C/30 s. A melting curve was generated by denaturing (95°C/10 s), then holding the reaction for 5 s at a temperature between 65°C and 95°C in 0.5°C increments. The template cDNA was diluted 10-fold in sterile water to an approximate concentration of 50 ng/µl. qPCRs were set up, measured and analyzed as described earlier (Witzel et al., 2018). Statistical analysis of transcript abundance data was assessed using Student’s t-test implemented in SigmaPlot 12.3 (Systat Software GmbH, Germany).

### Protein Extraction and Label Free Protein Quantification

Proteins in shoots and roots of the four *B. oleracea* genotypes were extracted using phenol extraction method (Faurobert et al., 2007). Resulting protein pellets were solubilized in 30 mM Tris, 7 M urea, 2 M thiourea, 4% CHAPS (pH 8.5), and concentration was measured using Bradford Red reagent (Expedeon, United Kingdom) and bovine serum albumin as standard. For tryptic digest of proteins, the filter aided sample preparation (FASP) protocol was applied as outlined in detail (Jozefowicz et al., 2018), using a Microcon-10 kDa Centrifugal Filter Unit (Merck Millipore, United States). After completion of digest, peptides were eluted from the filter and the eluate was dried. Desalting of peptides was done using Peptide Desalting Spin Columns (Pierce, Thermo Scientific, United States) following the manufacturer’s instructions. Eluted and desalted peptides were re-suspended in 2% acetonitrile/0.1% trifluoroacetic acid to a concentration of 100 ng µl^-1^.

Five µl of protein digest were separated by nanoflow liquid chromatography on a Dionex UltiMate 3000 system (Thermo Scientific) coupled to a Q Exactive Plus mass spectrometer (Thermo Scientific). Peptides were loaded onto a C18 trap-column (0.3 × 5 mM, PepMap100 C18, 5 µm, Thermo Scientific) and then eluted onto an Acclaim PepMap 100 C18 column (0.075 x 150 mM, 3 µm, Thermo Scientific) at a flow rate of 300 nl min^-1^. The mobile phases consisted of 0.1% formic acid (solvent A) and 0.1% formic acid in 80% ACN (solvent B). Peptides were separated chromatographically by a 70 min gradient from 2% to 44% solvent B, with the column temperature set at 40°C. For electrospray ionization of peptides, a Nanospray Flex ion source was used, with spray voltage set at 1.80 kV, capillary temperature at 275°C, and S-lens RF level at 60. Mass spectra were acquired in positive ion and data-dependent mode. Full-scan spectra (375 to 1,500 m/z) were acquired at 140,000 resolution and MS/MS scans (200 to 2,000 m/z) were conducted at 17,500 resolution. Maximum ion injection time was 50 ms for both scan types. The 20 most intense MS ions were selected for collision-induced dissociation fragmentation. Singly charged ions and unassigned charge states were rejected; dynamic exclusion duration was set to 45 s.

The raw files were processed using Proteome Discoverer v2.3.0.523 (Thermo Scientific) and Mascot search engine v2.5.1.1 (Matrix Science Inc, United States), searching the *B. oleracea* protein database (release 42, http://plants.ensembl.org/Brassica_oleracea/Info/Index). The false discovery rate was set to 0.01 for proteins and peptides. Further parameters for database search were: peptide tolerance, 10 ppm; fragment ion tolerance, 0.02 Da; tryptic cleavage with max. 2 missed cleavages; carbamidomethylation of cysteine as fixed modification and oxidation of methionine as variable modification. The result lists were filtered for high confident peptides and their signals were mapped across all LC-MS experiments (four *B. oleracea* genotypes x two analyzed organs x three independent experiments x three replicate measurements = 72 LC-MS experiments) and normalized to the total peptide amount per same LC-MS experiment. Only unique peptides were selected for quantification and abundances of all peptides allocated to a specific protein were summed and compared. Statistical analysis of protein abundance data was assessed using Student’s t-test implemented in SigmaPlot 13.0 (Systat Software GmbH, Germany).

### Plasmid Construction

Due to the high sequence similarity of *BoESP* isoforms, cloning of specific full-length cDNA fragments from *B. oleracea* was not successful. Therefore, cDNAs were commercially synthesized (Eurofins Genomics Germany GmbH, Germany) and subsequently PCR amplified using the primers listed in **Table S2**. The resulting fragments were inserted into the pENTR-D/TOPO vector according to the manufacturer’s instructions (Thermo Fisher Scientific, Germany) and verified by sequencing. Subsequently, the individual ESP coding regions were recombined into the technique-specific destination vector, using L/R-Clonase (Thermo Fisher Scientific). To generate constructs for *A. thaliana* transformation, the destination vector pRB-35S-3xmyc (Bartetzko et al., 2009) was used while *E. coli* expression constructs were based on a Gateway^®^-compatible version of pMal-C2 (New England Biolabs GmbH, Germany).

### Protein Expression and Purification

For *in vitro* ESP assays, MBP-BoESP1, MBP-BoESP2, and MBP-BoESP3 were individually expressed in *E. coli* BL21 cells at 37°C for 3 h after induction with 0.5 mM IPTG. Bacteria were harvested, lysed by sonication and after centrifugation, the recombinant proteins were purified using amylose resin (New England Biolabs GmbH, Germany) according to the manufacturer’s instructions. The purity of the proteins was approximately 90% as analyzed by SDS-PAGE and Coomassie Blue staining.

### Generation of Stable *Arabidopsis thaliana* Transformants Expressing BoESP1-3

*A. thaliana* ecotype Hi-0 was transformed using the floral dip method (Clough and Bent, 1998). For the selection of transgenic plants, seeds of T0 plants were sterilized and sown onto Murashige and Skoog medium supplemented with Gamborg’s vitamin solution (1:1,000) and 50 µg ml^-1^ kanamycin. Primary transformants were allowed to self-fertilize and then propagated into the T2 generation.

### Western Blotting

Immunoblotting analysis and membrane staining was performed as described previously (Witzel et al., 2017). Ten µg of protein were separated on SDS gels (SERVAGel™ TG PRiME™ 8–16%, Serva, Germany). The blots were probed with the anti-c-myc-peroxidase conjugate antibody (Roche, Mannheim, Germany) in a dilution of 1:1,000. Immunodetection was carried out using Pierce ECL Western Blotting Substrate (Thermo Fisher Scientific, USA) and Octoplus QPLEX Fluorescence Imager (NH DyeAGNOSTICS, Germany). After image capture, total protein load was assessed by staining the blots using amido black 10B staining method (Witzel et al., 2017).

### Determination of ESP Activity

#### BoESP Activity With Pure GLS Standards

To compare the substrate specificities of the three recombinant BoESPs, the protocol of (Matusheski et al., 2004) was modified and adapted. In order to maximize GLS hydrolysis, vitamin C was added and Fe^2+^ concentration in the assay was reduced to 0.2 mM to optimize ITC formation while maintaining ETN formation from alkenyl GLS in the assay: Briefly, 50 µl of purified ESP (containing in total 6.5 µg ESP as determined by Bradford assay (Bradford, 1976), 350 µl of a 50 mM sodium acetate buffer (pH 5.5) containing 1 mM dithiothreitol and 0.2 mM of FeSO_4_, 10 µl of a 25.5 mM vitamin C solution, 50 µl of 0.5 U ml^-1^ myrosinase and 50 µl of a 10 mM solution of the GLS to be tested were mixed in an extraction tube and incubated for 1 h at room temperature. Then, hydrolysis products of the respective GLS were extracted and quantified according to the protocol described above. Water controls (without ESP) were analyzed as well for each GLS.

ESP activity was expressed as the % of ETN [or nitrile (CN) formation for nonalkenyl GLS]: % ETN = [ETN]/([ETN]+[ITC]+[CN])*100%; % CN = [CN]/([CN] + [ITC]) *100%. Each analysis consisted of three independent experiments (freshly purified ESP extract) that consisted of 2–3 technical replicates.

#### ESP Activity in ***B. oleracea*** Tissues

In order to analyze the ESP activity of *B. oleracea* root and shoot samples, ESP-rich plant extracts, prepared as described previously (Hanschen et al., 2018a) were used for the assays. The protocol described above was used in a slightly modified way: 50 µL of ESP-rich plant extract were mixed with 350 µl of the sodium acetate buffer containing dithiothreitol and FeSO_4_, 10 µl of the vitamin C solution, 50 µl of myrosinase solution and finally 50 µl of 5 mg ml^-1^ solution of allyl GLS were added and samples were incubated and analyzed as described above. As some of the ESP-extracts already could contain CETP, water controls were analyzed as well, by adding water instead of allyl GLS. These values were used to correct the sample values. The % of CEPT on all allyl GLS hydrolysis products was calculated (% CETP = [CEPT]/([CEPT]+[Allyl-ITC]+[Allyl-CN])*100%).

### Influence of pH on ESP Activity

In order to investigate the influence of pH value on allyl GLS hydrolysis by the three ESPs, the ESP assay described above was performed, except that the pH value of the sodium acetate buffer was set to pH 4, pH 5, pH 6, and pH 7. Three technical replicates were analyzed.

### Statistical Analysis

**Figure 1:**
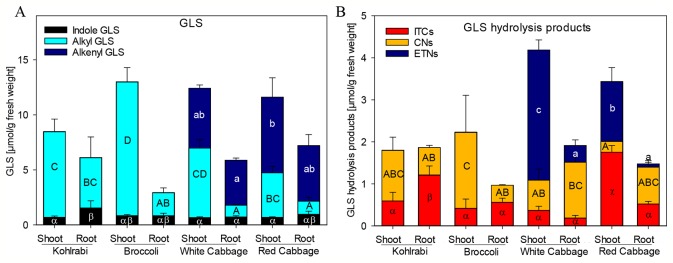
Glucosinolate (GLS) accumulation **(A)** and formation of hydrolysis products **(B)** in shoots and roots of four *B. oleracea* genotypes. ITCs, isothiocyanates; CNs, nitriles; ETNs, epithionitriles. Values represent mean ± standard deviation (SD) of three independent experiments (n = 3). Significant differences in means between the formation of), alkenyl GLS (small letters), alkyl GLS (capital letters), and indole GLS (Greek letters) in **(A)** or ETNs (small letters), CNs (capital letters), or ITC (Greek letters) in **(B)** as tested by ANOVA and Tukey HSD test at the p ≤ 0.05 level.

**Figure 2:**
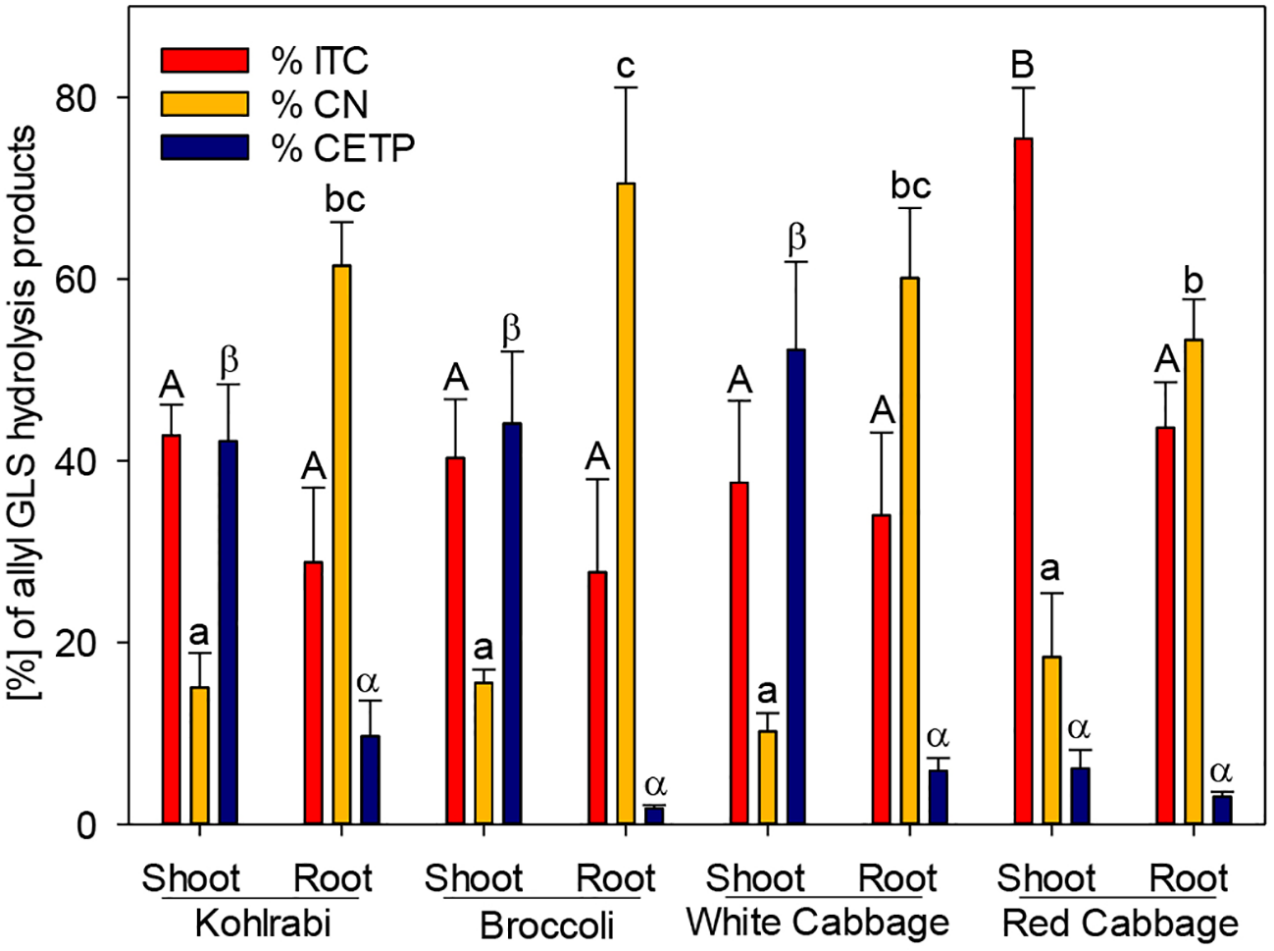
The effect of epithiospecifier protein activity of shoots and roots of four *B. oleracea* genotypes on the proportion of 1-cyano-2,3-epithiopropane (CEPT), the corresponding isothiocyanate (ITC) and nitrile (CN) produced from allyl glucosinolate (GLS). Values represent mean ± standard deviation of three independent experiments (n = 3). Significant differences in means between the formation of ITC (capital letters) or CN (small letters) or CETP (Greek letters) as tested by ANOVA and Tukey HSD test at the p ≤ 0.05 level.

To investigate statistical significant differences, one-way analysis of variance (ANOVA) was performed. For the comparison of means, Tukeys HSD test was applied using the STATISTICA version 13 software (StatSoft, Hamburg, Germany) (data in **Figures 1**, **2** and **7**, **Figure S2**) or the SigmaPlot version 13.0 (Systat Software GmbH, Germany) (data in **Figures 4**, **5** and **6**).

## Results

### Glucosinolate and Glucosinolate Hydrolysis Pattern in *B. oleracea* Genotypes

Previously, we have shown that *Brassica* vegetables exhibit a huge variation in the pattern of intact and hydrolyzed GLS (Hanschen and Schreiner, 2017; Klopsch et al., 2018). In order to characterize the plant material with regard to those specific plant secondary compounds, seedlings of four *B. oleracea* varieties, namely broccoli, kohlrabi, white cabbage, and red cabbage were investigated for their GLS profile and GLS hydrolysis product formation in roots and shoots. Both, broccoli and kohlrabi where rich in methylsulfanyl- and methylsulfinylalkyl GLS, but contained no alkenyl GLS (**Figure 1A**, **Table S3**). While shoot tissue was rich in the methylsulfinylalkyl GLS, 3MSOP, and 4MSOB, roots contained mainly the less oxidized 3MTP and 4MTB as well as 1-methoxyindol-3-ylmethyl GLS (**Table S3**). Shoots and roots of white and red cabbage accumulated alkenyl GLS (**Figure 1A**): allyl GLS in white cabbage and 2OH3But in red cabbage. Further, methylsulfanylalkyl GLS were present and shoots were rich in methylsulfinylalkyl GLS. Here, white cabbage contained mainly 3MSOP, whereas red cabbage was rich in 4MSOB. Additionally, white cabbage shoots contained 2PE (**Table S3**).

To screen for GLS hydrolysis products, the GLS in these plant genotypes and tissues were degraded upon endogenous enzymatic hydrolysis and corresponding nitriles, ITCs and ETNs (from alkenyl GLS) were produced (**Figure 1B**, **Table S4**). Nitriles were formed mainly in shoots of kohlrabi and broccoli, while roots released mainly ITCs; however, no ETNs was found in kohlrabi and only very low levels of ETNs (below 0.3%) were detected in broccoli (**Table S4**). Most abundant in white cabbage shoots were ETNs with 74% of degradation products, while roots formed mainly nitriles (70%) and less ETNs (20%). Shoots of red cabbage released mainly ITCs (51%), followed by ETNs (41%) and root tissue released mainly nitriles (59%) and low amounts of ETNs (5%). Of note is that, compared to total GLS contents, the overall recovery of GLS hydrolysis products was relatively low with 18–46%. The recovery of products from allyl GLS ranged from 39–63%, while that of the products of sulfinylalkyl GLS, 3MSOP and 4MSOB, was low with only 13%.

### ESP Activity in *B. oleracea* Is Genotype- and Organ-Specific

While ESP activity *via* plant tissue autolysis assay was detected only in white and red cabbage, an additional assay was performed to analyze plant tissue ESP activity on allyl GLS. The potential of the tested genotypes to form ETN as an indicator of ESP activity was assessed by studying the hydrolysis of allyl GLS to the corresponding ETN (1-cyano-2,3-epithiopropane, CETP) in presence of myrosinase. ESP activity of shoots was high in kohlrabi, broccoli, and white cabbage (42%, 44%, and 52%, respectively), while it was lower in shoots of red cabbage (**Figure 2**). ESP activity was in general inversely correlated to CN formation in this assay. Roots possessed a lower ESP activity as compared to shoots, but root tissue of kohlrabi had by tendency higher ESP activity than shoot tissue of red cabbage (9.7% vs. 6.1%). Shoot tissue of red cabbage significantly released more ITC than the other tissues in this assay (**Figure 2**). These results indicate that all *B. oleracea* genotypes and tissues exhibit ESP activity, including kohlrabi and broccoli.

### Identification of Genes in *B. oleracea* With Sequence Similarity to AtESP

**Figure 3:**
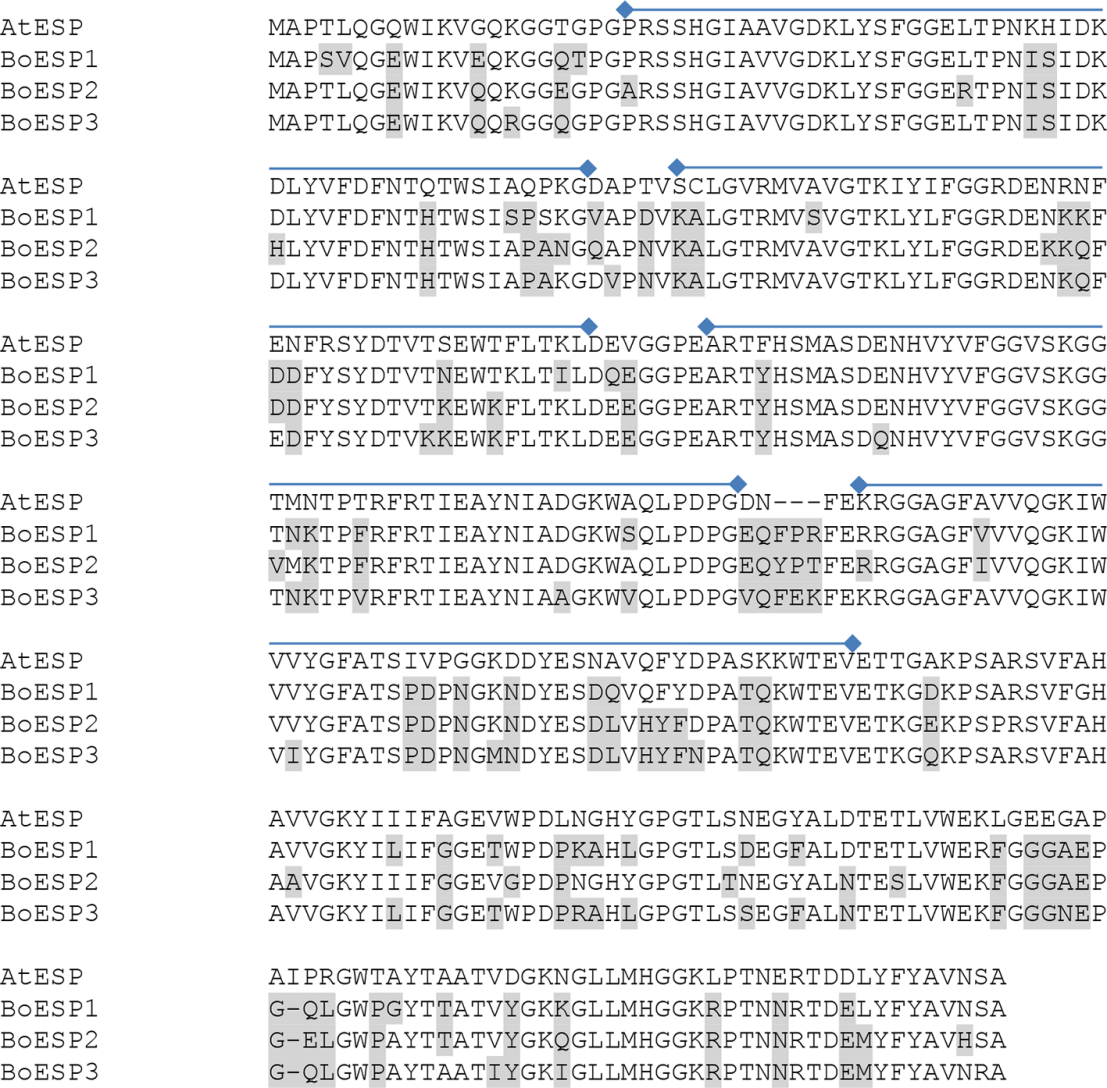
Alignment of the amino acid sequences of AtESP (At1g54040.2) and *Brassica oleracea* ESPs BoESP1 (LOC106296341), BoESP2 (LOC106306810), and BoESP3 (LOC106325105) using T-Coffee program (Tommaso et al., 2011). Residues variant from AtESP are shaded in grey. Kelch domains (PF01344, Kelch_1) of AtESP are indicated by lines above the alignment and were derived from pfam (http://pfam.xfam.org).

Based on our observation of differential ETN formation in the tested genotypes and plant organs, we hypothesized the presence of multiple ESP isoforms in *B. oleracea*. Sequence similarity search using *AtESP* nucleotide sequence (At1g54040.2) against the whole genome of *B. oleracea* revealed six predicted ESP-like sequences (**Table 1**). Amino acid sequence alignment showed that three of the predicted genes were almost identical: LOC106306810 (designated *BoESP2*), LOC106306884X1, and LOC106306884X2 (**Table 2**). Hence, these three predicted genes were considered as one isoform. The amino acid sequence of LOC106297542 was considerably shorter as compared to the remaining ESPs and shared only N-terminal sequence similarity to AtESP. Since the protein contained only two Kelch domains, this gene was omitted from further analysis. In order to test if the predicted genes are actively transcribed in shoots of the four *B. oleracea* genotypes, the open reading frames of the putative sequences were aligned and primers were designed to bind to their isoform-specific regions (**Table S1**, **Figure S1**). The specificity of amplification was verified by sequencing of PCR products. PCR products were obtained for LOC106296341 (designated *BoESP1*), LOC106306810 (designated *BoESP2*), and LOC106325105 (designated *BoESP3*) and, therefore, those three ESP isoforms were investigated further. Sequence comparison with *AtESP* showed that *BoESP1-3* had a sequence similarity between 79 and 81% at nucleotide level (**Figure S1**), and 77% on amino acid level (**Figure 3**).

**Table 1:** Predicted and characterized ESP-like genes present in *Brassica oleracea*.

Protein isoform	NCBI gene ID	NCBI protein ID	Ensembl Plants ID	Phytozome ID	ORF (aa)
BoESP1	LOC106296341	XP_013587912.1	Bo6g032960.1	Bol039072	343
BoESP2	LOC106306810	XP_013599019.1	Bo7g067530	Bol006380	343
BoESP3	LOC106325105	XP_013618586.1	Bo2g124420.1	Bol024137	343
–	LOC106297542	XP_013589216.1	Bo6g050860	Bol013374	158
–	LOC106306884X1, LOC106306884X2	XP_013599116.1, XP_013599117.1	Bo7g067500	Bol006378	343

The AtESP nucleotide sequence (At1g54040.2) was used to Blast *B. oleracea* entries of the NCBI database (https://www.ncbi.nlm.nih.gov), *B. oleracea* entries of the Ensembl Plants v43 database (https://plants.ensembl.org/index.html) and *B. oleracea* var. *capitata* v1.0 entries of the Phytozome v12.1 database (https://phytozome.jgi.doe.gov/pz/portal.html#).

**Table 2:** Amino acid sequence similarity of identified ESP-like genes found in *B. oleracea*, given as per cent identity.

LOC 106325105 (BoESP3)	LOC 106296341 (BoESP1)	LOC 106297542	LOC 106306810 (BoESP2)	LOC 106306884X1	LOC 106306884X2	
	86.0	79.1	86.3	86.6	86.6	**LOC106325105**
		73.2	84.5	84.8	84.8	**LOC106296341**
			77.1	77.8	77.8	**LOC106297542**
				99.7	99.7	**LOC106306810**
					100.0	**LOC106306884X1**
						**LOC106306884X2**

### Transcript and Protein Expression Pattern of BoESP1-3

**Figure 4:**
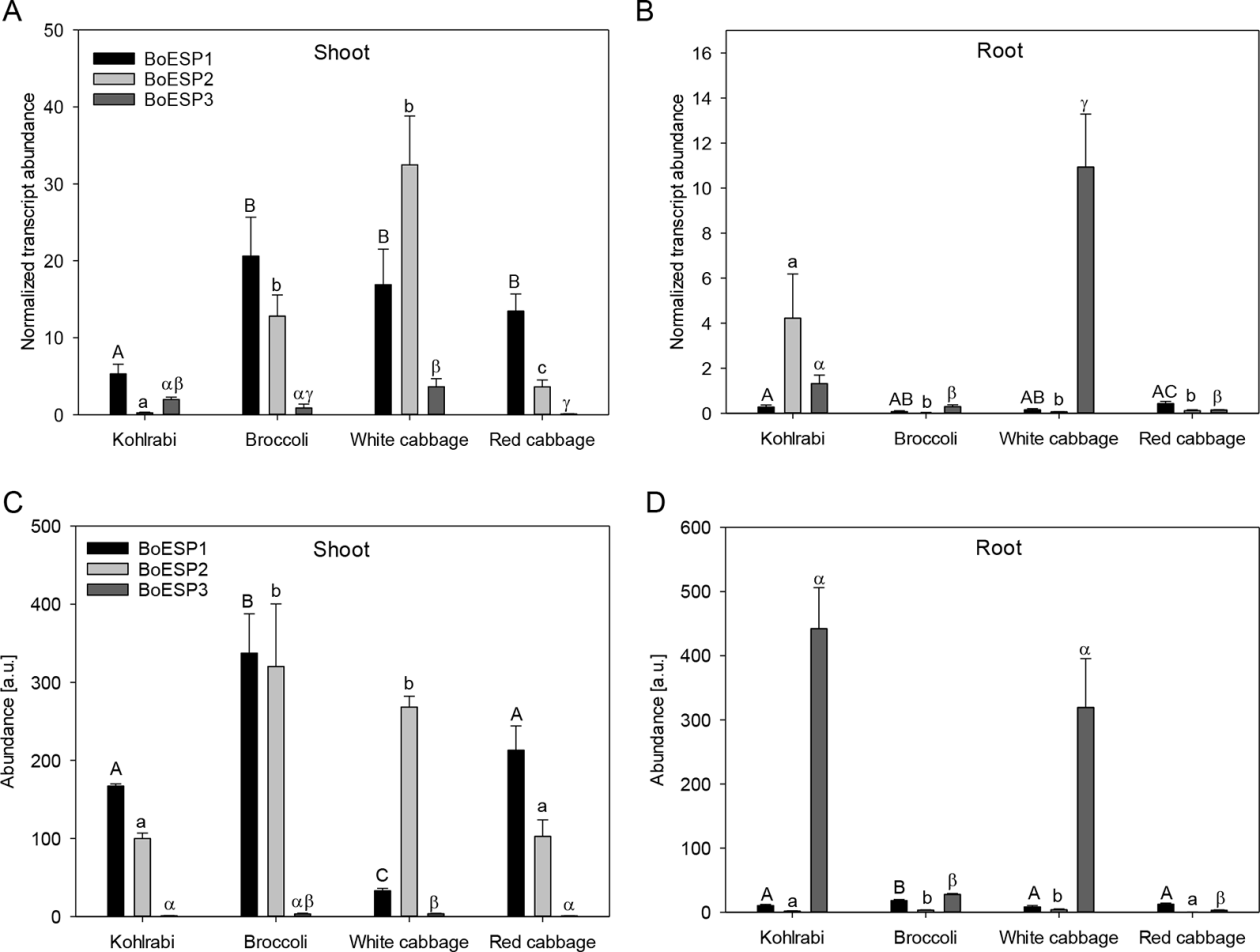
Expression profiles of BoESP1-3 transcripts **(A**, **B)** and proteins **(C**, **D)** in shoots and roots of four *B. oleracea* genotypes. Values represent mean ± standard error of measurements based on three technical replicates from four independent biological experiments for transcript abundance, and based on three technical replicate runs from three independent biological experiments for protein abundance. Letters denote statistical significant differences (p ≤ 0.05) of BoESP1 (capital letters), BoESP2 (small letters), or BoESP3 (Greek letters) between the genotypes within a given plant organ.

In order to analyze transcript abundance levels in shoot and roots of sprouts, quantitative real-time PCR was performed. The analysis revealed a higher expression level of *BoESP1* in shoots as compared to roots (**Figures 4A, B**). *BoESP2* was higher abundant in shoots of broccoli, white cabbage, and red cabbage as compared to the respective root samples, although this pattern was reversed in kohlrabi. *BoESP3* expression was barely detected in shoots, while a transcript accumulation was detected in roots of kohlrabi and white cabbage.

We further extended the expression analysis to profile protein abundance pattern based on label-free LC-MS analysis. A total of 20,896 peptide groups were identified by this approach in shoots and roots of the four genotypes, leading to the identification of 6,986 proteins, which were grouped into 3,996 protein groups based on sequence similarity. Then, the data was inspected for BoESP1-3 presence and abundance. Protein coverage was higher for BoESP1 (69%) and BoESP2 (75%) as compared to BoESP3 (42%). Nevertheless, a sufficient amount of isoform-specific peptides was detected allowing the quantification of isoforms based on peptides that were unique to the respective isoform. Nine unique peptides were found for BoESP1, 16 unique peptides were detected for BoESP2, and eight unique peptides were specific for BoESP3. Overall, the protein abundance pattern (**Figures 4C, D**) was in agreement with the transcript abundance. However, while transcript levels were threefold higher in shoots as compared to roots, the level of accumulation of the respective gene products was similar between both plant organs.

### Substrate Specificity of BoESP Isoforms

**Figure 5:**
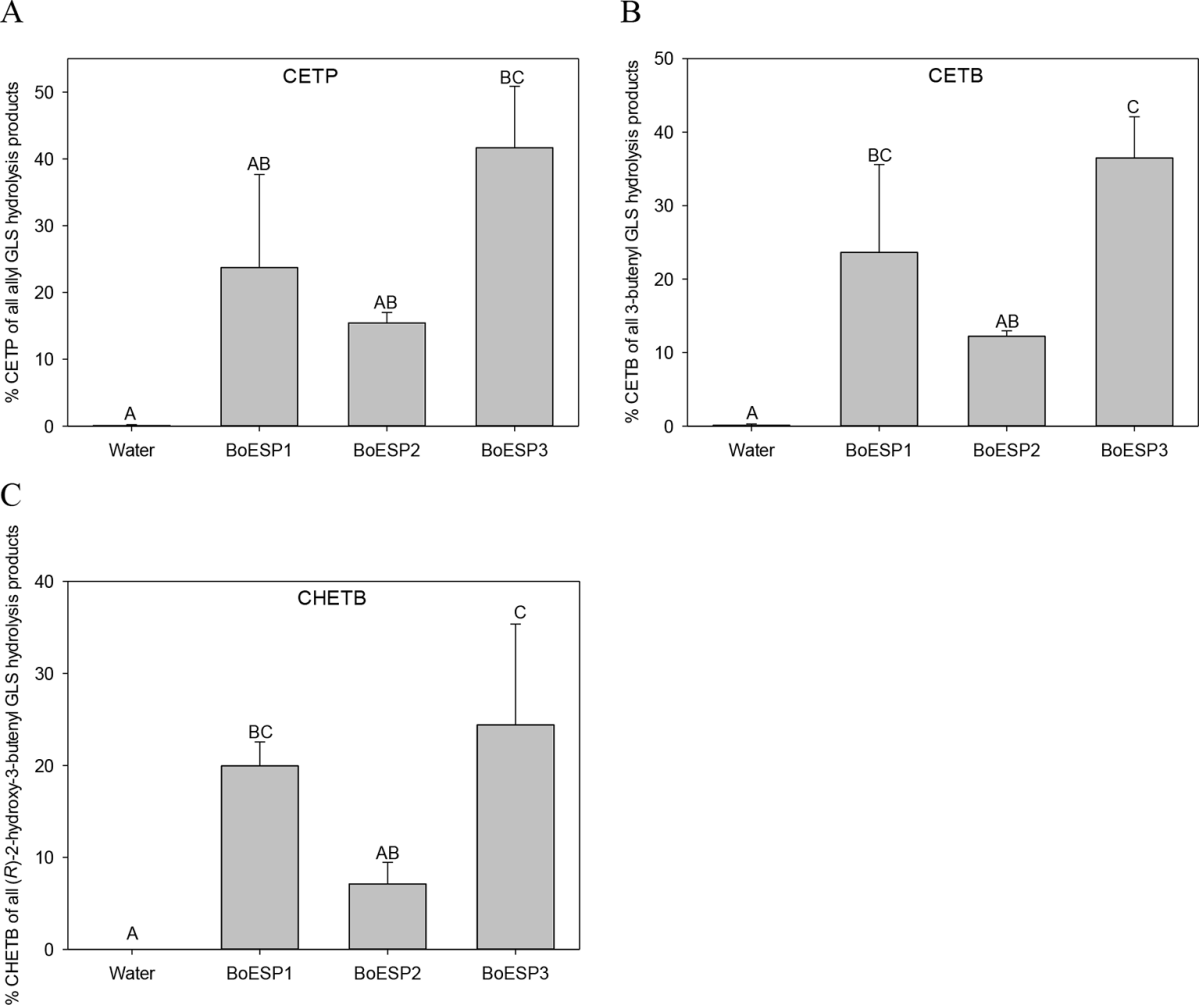
Glucosinolate (GLS) substrate-specific epithiospecifier protein (ESP) activity of recombinant BoESP1-3 as assessed by the formation of epithionitriles from the three GLS **(A)** allyl GLS, **(B)** 3-butenyl GLS and **(C)** (R)-2-hydroxy-3-butenyl GLS. CETP, 1-cyano-2,3-epithiopropane; CETB, 1-cyano-3,4-epithiobutane; CHETB, 1-cyano-2-hydroxy-3,4-epithiobutane. Values represent mean ± standard deviation of three independent experiments, comprising of 2–3 technical replicates each. Different capital letters indicate significant differences in means between the formation of the epithionitrile from one GLS by the different BoESP and a water control as tested by ANOVA and Tukey test at the p ≤ 0.05 level.

Since the three BoESP isoforms exhibited a differential expression pattern, their substrate specificity was tested next, using recombinant BoESP1-3 proteins. Open reading frames of BoESP1-3 were cloned, expressed in *E.coli* and proteins purified using the maltose-binding protein tag. The effect of the substrate structure on the activity of the three BoESPs was investigated using three alkenyl GLS, three alkyl GLS and one aromatic GLS. With all three alkenyl GLS investigated, namely allyl GLS (releasing CETP), 3But (releasing CETB) and 2OH3But (forming CHETB), BoESP3 showed the highest ESP activity, and BoESP2 (by tendency for allyl GLS hydrolysis) the lowest ESP activity (**Figure 5**). BoESPs did not increase nitrile release from the alkenyl GLS (**Table S5**). Moreover, there was no effect of the three BoESPs on the hydrolysis of nonalkenyl GLS [4MTB releasing 5-(methylsulfanyl)pentanenitrile (4MTB-CN), 3MSOP releasing 4-(methylsulfinyl)butanenitrile (3MSOP-CN), and 4MSOB releasing 5-(methylsulfinyl)pentanenitrile (4MSOB-CN)](**Figure S2**).

### The pH Affects BoESP Activity Differently

**Figure 6:**
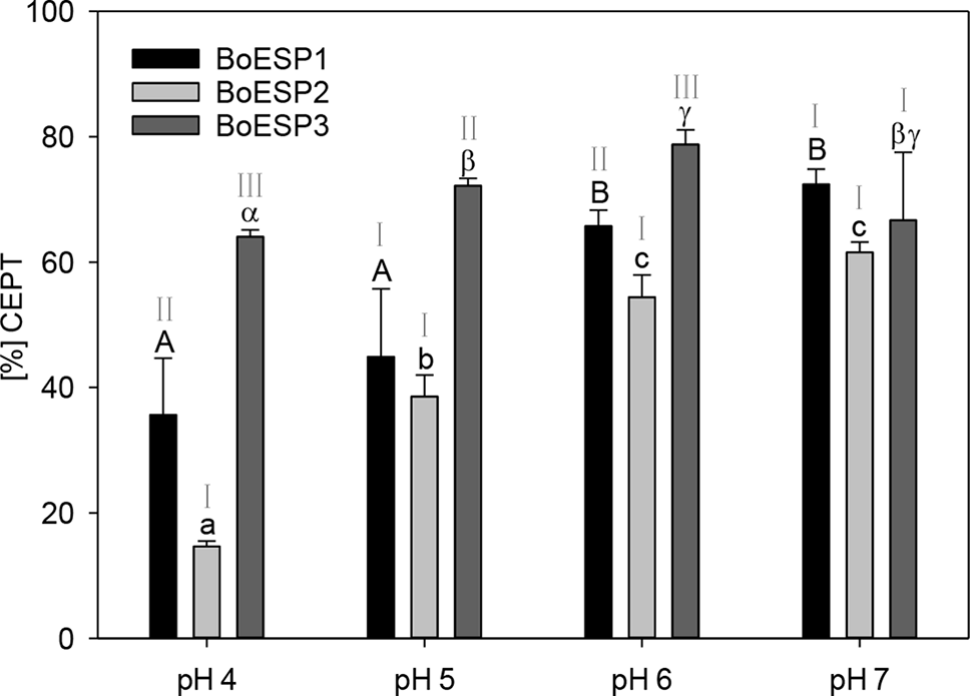
Influence of the pH value on the epithiospecifier protein (ESP) activity of BoESP1-3 assessed by the formation of 1-cyano-2,3-epithiopropane (CETP) from allyl glucosinolate (GLS). Values represent mean ± standard deviation of three replicate measurements. Different letters indicate significant differences in means for the influence of pH on CETP-formation by BoESP1 (capital letters), BoESP2 (small letters), or BoESP3 (Greek letters) as tested by ANOVA and Tukey test at the p ≤ 0.05 level or, in case of BoESP3, by pairwise comparison using t-test. Roman letters indicate significant differences in means between the formation of CETP by the different BoESP at a given pH value as tested by ANOVA and Tukey test at the p ≤ 0.05 level.

The pH-optimum can be used as a measure to assess protein adaptation to cellular and subcellular pH as well as to get insight into protein-protein interactions that take place in the same microenvironment (Talley and Alexov, 2010). The optimal pH value for *B. napus* (Bernardi et al., 2000) and *Crambe abyssinica* (Tookey, 1973) ESPs was reported to be pH 6, but so far the pH optimum for BoESP was not published. Therefore, the effect of pH values on the activity of the three BoESP was investigated in a pH range of pH 4 to pH 7 (**Figure 6**). While BoESP3 activity was high at all pH values, both BoESP1 and BoESP2 were affected. Being at optimal pH from 6–7, their activity decreased with decreasing pH value. This effect was stronger for BoESP2 compared to BoESP1, especially at pH 4.

### Expression of BoESP1-3 in *A. thaliana* Hi-0

**Figure 7:**
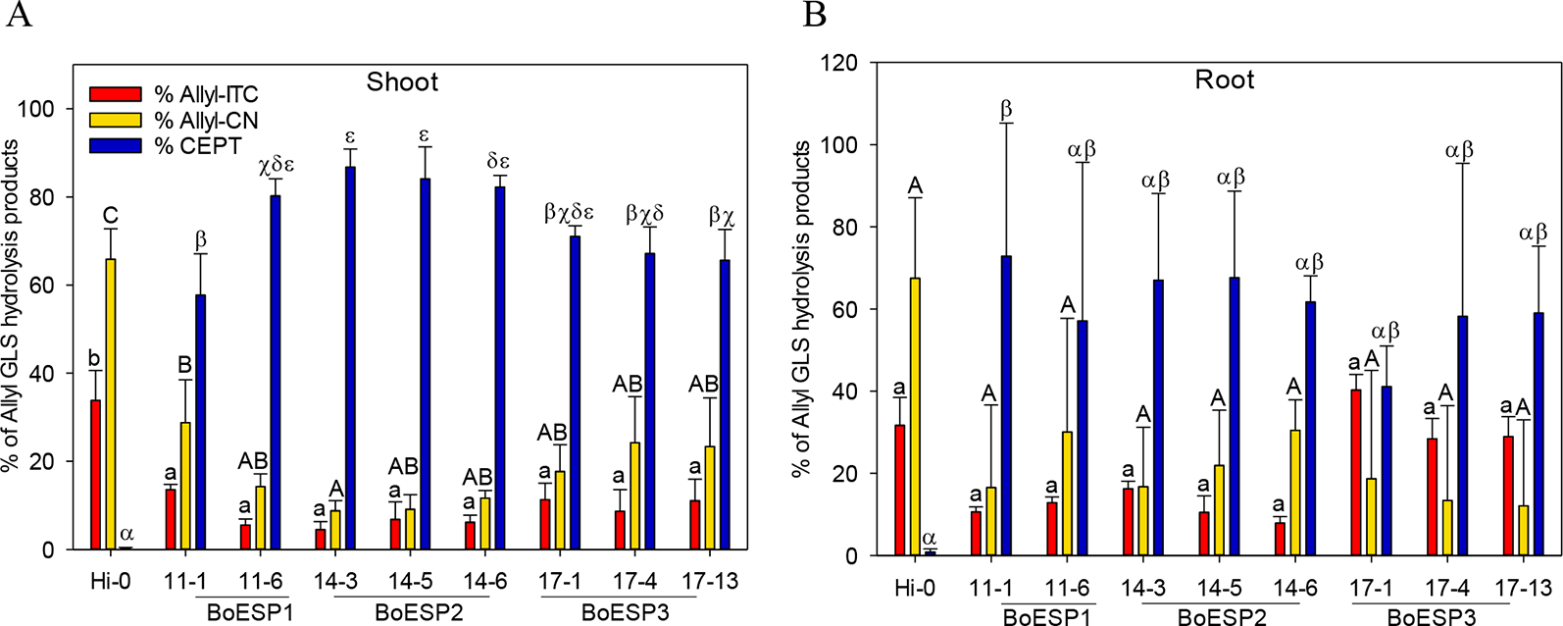
Formation of allyl GSL hydrolysis products allyl isothiocyanate (Allyl-ITC), 3-butenenitrile (Allyl-CN) and 1-cyano-2,3-epithiopropane (CETP) in % relative to all allyl GLS hydrolysis products in *Arabidopsis thaliana* Hi-0 control and BoESP1-3 transgenic lines in shoots **(A)** and roots **(B)**. Values represent mean ± standard deviation of three independent experiments. Significant differences in means between the formation of Allyl-CN (small letters), Allyl-ITC (capital letters) or CETP (Greek letters) as tested by ANOVA and Tukey HSD test at the p ≤ 0.05 level.

In order to test whether BoESP1-3 isoforms are able to perform ESP activity *in planta*, the three isoforms were expressed in *A. thaliana* Hi-0, an ecotype with a GLS pattern that is characterized by high allyl GLS content and barely detectable own ESP activity (Hanschen et al., 2018b). Two independent lines expressing cDNAs of BoESP1 and three independent lines expressing BoESP2 and BoESP3, respectively, were characterized in comparison to the Hi-0 wildtype. Western blot analyses using an antibody directed against the C-terminal myc-tag confirmed BoESP protein in all transgenic lines (**Figure S3**). Analysis of transgenic *A. thaliana* plants revealed a modified hydrolysis pattern of allyl GLS (**Figure 7**). Levels of released GLS hydrolysis products are given in the **Table S6**. Whereas Hi-0 wildtype plants released mainly Allyl-CN [1.09 ± 0.17µmol/g fresh weight (FW)], Allyl-ITC (0.56 ± 0.09 µmol/g FW), and very low CETP (0.006 ± 0.002 µmol/g FW) from allyl GLS in shoots and roots, BoESP1-3 transformants released mainly CETP from these tissues. Shoots of *A. thaliana* BoESP2 constructs showed a slightly higher percentage of CETP-formation compared to BoESP3 constructs. The shift in allyl GLS hydrolysis in shoot tissue was accompanied with reduced formation of both Allyl-ITC and Allyl-CN in the transgenic lines. Since the overall amount of allyl GLS in roots is 10-times lower as compared to shoots (Witzel et al., 2013), the detection of respective breakdown products in wildtype and transgenic lines was near the method detection limit, resulting in substantial standard deviations. Nonetheless, ESP activity was increased in BoESP1-3 transformants, as compared to the wildtype. The hydrolysis of the alkyl GLS 8-(methylsulfanyl)octyl (8MTO) in *A. thaliana* was not affected by the BoESPs (**Figure S4**).

## Discussion

GLS hydrolysis in *Brassica* vegetables often results in the release of nitriles and ETN instead of ITCs, and presence of ESP is made responsible (Petroski and Tookey, 1982; Matusheski et al., 2006; Hanschen and Schreiner, 2017; Klopsch et al., 2018). Here, we report the identification and characterization of three BoESP isoforms in *B. oleracea* varieties, namely kohlrabi, broccoli, white cabbage, and red cabbage and their functional characterization in *A. thaliana*.

GLS hydrolysis in root and shoot tissue of four *B. oleracea* genotypes was investigated and was correlated with ESP activity in order to characterize the role of ESP in the GLS hydrolysis. The genotypes that contained no alkenyl GLS (kohlrabi, broccoli) were producers of nitriles, while the genotypes containing alkenyl GLS (white cabbage, red cabbage) formed ETN in high amounts, which is in line with previous reports (Matusheski et al., 2006; Hanschen and Schreiner, 2017; Hanschen et al., 2018a). In contrast to this, the ESP activity assay revealed that all genotypes and tissues possess ESP activity. This is mirrored by the high ETN release in alkenyl rich genotypes (white cabbage, red cabbage) or high nitrile release in shoot tissue of alkyl GLS rich genotypes and points to the dual role of ESPs in generating nitriles as well as ETNs (Lambrix et al., 2001), depending on the GLS substrate.

Our analysis identified six genes in the *B. oleracea* genome with sequence homology to *AtESP*. Screening the transcript abundance of these genes revealed that three of them were expressed in shoots of seedlings. It remains open whether those genes, where no expression was detected, are transcribed at older developmental stages, in other tissues or genotypes as investigated, have lost their function or are induced under specific environmental conditions. One of the expressed genes, *BoESP1*, was identical to a previously characterized ESP from *B. oleracea* (Matusheski et al., 2006). Expression of *BoESP*s was regulated differentially with respect to the analyzed tissue and the investigated genotypes. *BoESP1* and *BoESP2* were detected mainly in shoots, while *BoESP3* was found in shoots but accumulated more in roots, confirming previous data from RNAseq experiments (Wang et al., 2017). The presence of *BoESP3* in roots distinguishes it from *AtESP*, for which only low transcript levels and no protein abundance were detected in this plant organ (Burow et al., 2007; Kissen et al., 2012). In general, pattern of transcripts were reflected by those of protein abundances. However, the overlap between protein expression pattern (**Figure 4**) and GLS hydrolysis generation (**Figures 1** and **2**) was limited to the shoot. In roots, although BoESP3 expression was high in kohlrabi and white cabbage, white cabbage produced nitriles and ETNs from endogenous GLS, while kohlrabi produced only small amounts of nitriles (**Figure 1**). In addition, the hydrolysis pattern of allyl GLS was almost identical between genotypes with high BoESP3 expression in roots (kohlrabi and white cabbage) and those with barely detected BoESP3 abundance (broccoli and red cabbage) (**Figure 2**). These observations could indicate the presence and functionality of other specifier proteins that are not elucidated yet. This research question is particularly interesting since roots were mainly excluded from past investigations on GLS degradation, although GLS and GLS degradation products were detected in root exudates and might play a role in below ground chemical communication between plants and rhizosphere biota (Schreiner et al., 2011; Auger et al., 2012; Xu et al., 2017). Moreover, future research should be directed to enlighten the cellular and subcellular localization of BoESP1 and BoESP2, which would explain their biological role in more detail. RNAseq data from *B. oleracea* indicate that BoESP2 is highly expressed in leaves, while BoESP1 expression peaks in stems, flowers, and siliques (Wang et al., 2017).

The recombinant BoESPs differed in their activity towards alkenyl GLS, with BoESP3 being most active and BoESP2 being least active under the assay conditions. In contrast, *A. thaliana* lines expressing BoESP2 showed the highest percentage of ETN release during GLS hydrolysis. The activity of ESPs is strongly dependent on Fe^2+^ (Backenköhler et al., 2018) and probably differs in their requirement of Fe^2+^ as previous reports suggest (Williams et al., 2010). Therefore, it is likely that BoESPs activities will vary according to hydrolysis conditions present.

The recombinant BoESPs showed no activity in terms of the hydrolysis of the tested non-alkenyl GLS, implying that these proteins do not possess NSP activity (**Figure S2**) as this would have been linked with the increased formation of simple nitriles (Wittstock et al., 2016). This corresponds to the hydrolysis pattern of alkyl GLS 8MTO, observed in the *A. thaliana* transgenic lines (**Figure S4**). An absent NSP activity of recombinant AtESP on 4MSOB GLS substrate was reported earlier (De Torres Zabala et al., 2005). However, NSP activities of ESPs on alkyl GLS have been observed by other groups (Burow et al., 2006; Matusheski et al., 2006) and the tested non-alkenyl GLS are hydrolyzed to nitriles *in vivo* in the tested genotypes (**Table S2**). Hence, our results could indicate the presence of NSPs in *B. oleracea* that have not been characterized yet. The gene expression of two NSP in the related *B. rapa* species was already reported (Wang et al., 2017). Interestingly, the *Pieris rapae*NSP activity was differently affected by Fe^2+^ concentrations. More 4MTB-CN was released in presence of ESP from 4MTB (13-fold of ESP free control) when Fe^2+^ was absent as compared to 0.01 mM Fe^2+^, while more 4MSOB-CN was formed at the 0.01mM Fe^2+^ level as compared to no added Fe^2+^ (Burow et al., 2006).

Moreover, as tested here, the BoESPs differed in their susceptibility towards a shifting pH. BoESP1 and BoESP2 activity decreased with a lower pH, whereas the activity of BoESP3 was stable in a pH range of pH 4 to pH 7. This stability of BoESP3 could point to a different subcellular localization as compared to BoESP1 and BoESP2. Cellular compartmentation is crucial to control the complex system of GLS hydrolysis. GLS accumulate in vacuoles of sulfur-rich S-cells that are found in close proximity of myrosin cells and guard cells, which both store myrosinases in their vacuoles (Shirakawa and Hara-Nishimura, 2018). In contrast to this, AtESP is localized in the cytosol (Burow et al., 2007; Chhajed et al., 2019). Measurements of the *in vivo* organelle pH revealed an acidification gradient from the cytosol and endoplasmatic reticulum, with pH around pH 7, to lytic vacuoles ranging from pH 5–6 (Martinière et al., 2013; Shen et al., 2013). The pH of the leaf apoplast is even more acidic and was determined to range from 4.5–5 (Geilfus and Muehling, 2011). Hence, one possible explanation for the pH stability of BoESP3 could be the additional localization to the root apoplast. So far, apoplast proteome studies demonstrated the presence of myrosinase and myrosinase-associated proteins in *B. juncea* (Sehrawat and Deswal, 2014) and *A. thaliana* (Trentin et al., 2015) leaves, but not for ESPs. Since BoESP3 is expressed mainly in roots, future experiments should concentrate on the subcellular localization of this isoform to further study its possible involvement in root exudation of GLS hydrolysis products.

The general role of specifier proteins *in planta* is still part of scientific debate. In *Arabidopsis*, the NSP-catalyzed release of simple nitriles often increases due to herbivory (Lambrix et al., 2001; Burow et al., 2009). Moreover, ESP/NSP-expressing nitrile-producing plants are more susceptible to herbivore feeding (Lambrix et al., 2001; Burow et al., 2009; Jeschke et al., 2016). However, increased nitrile formation can reduce attractiveness for ovipositing herbivorous specialist insects and attracts natural enemies of the larvae (Mumm et al., 2008; Jeschke et al., 2016). So far, the specific role of ESP-catalyzed ETN formation and, hence, their specific role in biotic interactions is still unclear (Jeschke et al., 2016). With regard to human consumption, the aroma and taste of fresh Brassicaceae vegetables likely is influenced by the ESP activity (Zhang et al., 2018). While ITC contribute to the characteristic flavor and pungency of these vegetables (Bell et al., 2018), ETNs have a lower aroma impact. Using GC-olfactometry, the overall odor impression of the ETN CETP was described as weak and onion-like, pungent, spicy, fatty, but also garlic-like and fecal notes were attributed to this compound (Kroener and Buettner, 2017). Therefore, a higher ESP activity might reduce the taste and flavor of fresh *Brassica* vegetables. Further, vegetables rich in ESP-activity might have lower health beneficial properties, as protective effects are mainly attributed to ITCs (Zhang et al., 2018). Thus, it is crucial to unravel the role of ESP *in planta* and to identify strategies for ESP activity reduction in these vegetables.

## Data Availability Statement

All datasets generated for this study are included in the article/**Supplementary Material**.

## Author Contributions

FH and KW designed the study. FH, KW, MA, and PA performed the experiments. FH, MA, FB, and KW analyzed the data. FH and KW wrote the manuscript, with contributions from all authors.

## Funding

FH is funded by the German Leibniz Association (Leibniz-Junior Research Group OPTIGLUP; J16/2017).

## Conflict of Interest

The authors declare that the research was conducted in the absence of any commercial or financial relationships that could be construed as a potential conflict of interest.
