# Corrigendum: Identification and Characterization of Three Epithiospecifier Protein Isoforms in *Brassica oleracea*

**DOI:** 10.3389/fpls.2020.00523

**Published:** 2020-04-28

**Authors:** Katja Witzel, Marua Abu Risha, Philip Albers, Frederik Börnke, Franziska S. Hanschen

**Affiliations:** ^1^Leibniz Institute of Vegetable and Ornamental Crops, Großbeeren, Germany; ^2^Institute of Biochemistry and Biology, University of Potsdam, Potsdam, Germany

**Keywords:** epithionitrile, expression profile, functional complementation, glucosinolate hydrolysis, nitrile, specifier proteins, tissue specificity

In the original article, there was a mistake in Figure 1 as published. In Figure 1A as well as in Figure 1B the colors in the bar of the “Red Cabbage Root” were not shown correctly: In Figure 1A the “Indole GLS” in the bar of “Red Cabbage Root” were turquois instead of black. In Figure 1B in the bar of “Red Cabbage Root” the ITCs were orange instead of red and the CNs were dark blue instead of orange (and therefore could be mistaken for ETNs). The corrected Figure 1 appears below.

**Figure 1:**
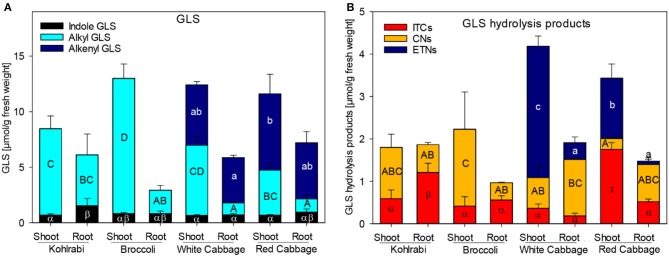
Glucosinolate (GLS) accumulation **(A)** and formation of hydrolysis products **(B)** in shoots and roots of four *B. oleracea* genotypes. ITCs, isothiocyanates; CNs, nitriles; ETNs, epithionitriles. Values represent mean ± standard deviation (SD) of three independent experiments (n = 3). Significant differences in means between the formation of), alkenyl GLS (small letters), alkyl GLS (capital letters), and indole GLS (Greek letters) in **(A)** or ETNs (small letters), CNs (capital letters), or ITC (Greek letters) in **(B)** as tested by ANOVA and Tukey HSD test at the p ≤ 0.05 level.

The authors apologize for this error and state that this does not change the scientific conclusions of the article in any way. The original article has been updated.

