# Supplementary material for: Identification and Characterization of Three Epithiospecifier Protein Isoforms in Brassica oleracea
Source: Front Plant Sci. 2019 Dec 19;10:1552. doi: 10.3389/fpls.2019.01552 (PMC6930892; doi:10.3389/fpls.2019.01552)
Supplement: Supplementary file 1 [file Image_1.pdf]

Figure S1: Alignment of the nucleotide sequences of *Brassica oleracea* ESPs. Residues variant from the consensus are shaded in grey. Underlined nucleotides represent the position of primer sequences, while residues colored in red indicate respective forward primer sequences and residues colored in blue show the position of reverse primers.

```
BoESP1          ATGGCTCCGAGTGTGCAAGGCGAGTGGATCAAGGTCGAGCAGAAAGGAGG
BoESP2          ATGGCTCCCACATTGCAAGGCGAGTGGATCAAGGTGCAGCAGAAAGGAGG
BoESP3          ATGGCTCCGACTTTGCAAGGCGAGTGGATCAAGGTGCAGCAGAGAGGAGG

BoESP1          ACAGACACCAGGACCGAGAAGCTCACATGGCATAGCCGTGGTCGGAGACA
BoESP2          AGAGGGACCTGGAGCAAGAAGCTCACACGGCATAGCCGTGGTCGGAGACA
BoESP3          ACAAGGACCTGGACCAAGAAGCTCACACGGCATAGCTGTGGTTGGAGACA

BoESP1          AGCTCTACTCGTTTGGTGGCGAGTTAACTCCCAACATTTCCATCGACAAA
BoESP2          AGCTCTACTCTTTCGGTGGCGAGCGTACGCCAAACATTTCCATCGACAAA
BoESP3          AGCTCTATTCTTTCGGCGGGGAGCTGACCCCAAACATTTCCATCGACAAA

BoESP1          GACCTTTACGTCTTTGACTTCAACACTCACACTTGGTCAATCTCTCCGTC
BoESP2          CACCTTTACGTCTTTGACTTCAACACTCACACTTGGTCAATCGCCCCGGC
BoESP3          GACCTTTACGTCTTTGACTTCAACACTCACACTTGGTCAATCGCTCCAGC

BoESP1          CAAGGGAGTAGCCCCTGACGTCAAGGCCTTGGGCACCCGCATGGTGTCCG
BoESP2          CAACGGACAAGCCCCTAACGTCAAGGCCCTGGGCACCCGCATGGTGGCCG
BoESP3          CAAGGGAGACGTTCCTAACGTCAAGGCCTTGGGCACCCGCATGGTGGCTG

BoESP1          TGGGAACTAAGCTCTATCTATTCGGAGGCCGCGACGAGAATAAAAAGTTC
BoESP2          TGGGAACTAAGCTCTATCTATTCGGAGGCCGCGATGAGAAGAAACAGTTC
BoESP3          TTGGAACTAAGCTCTATCTCTTCGGAGGCCGCGATGAGAATAAACAATTT

BoESP1          GATGACTTTTATTCGTACGATACGGTGACAAATGAATGGACAAAACTGAC
BoESP2          GACGACTTTTACTCGTACGATACAGTGACAAAGGAGTGGAAGTTCCTCAC
BoESP3          GAGGACTTTTATTCATACGATACCGTGAAAAAGGAGTGGAAGTTCCTGAC

BoESP1          CATTCTGGATCAAGAGGGAGGACCCGAGGCTCGAACTTACCACTCCATGG
BoESP2          CAAGCTCGATGAAGAGGGAGGACCTGAGGCTCGTACTTACCACTCGATGG
BoESP3          GAAGCTCGATGAAGAGGGAGGACCTGAGGCCCGCACTTACCATTCGATGG

BoESP1          CTTCGGATGAAAACCATGTGTATGTATTCGGTGGAGTGAGCAAAGGAGGG
BoESP2          CTTCGGATGAAAACCACGTGTATGTATTCGGCGGGGTGAGCAAAGGCGGG
BoESP3          CTTCCGATCAAAACCATGTGTATGTGTTCGGTGGAGTGAGCAAAGGTGGG

BoESP1          ACCAACAAGACACCCTTTAGGTTCAGGACCATCGAGGCCTATAACATTGC
BoESP2          GTTATGAAGACCCCCTTTAGGTTCAGGACCATCGAGGCGTATAACATTGC
BoESP3          ACGAACAAAACCCCTGTGCGTTTCAGGACGATCGAGGCCTATAACATTGC

BoESP1          TGATGGGAAATGGTCTCAGCTTCCTGATCCTGGTGAGCAGTTCCCAAGGT
BoESP2          TGATGGGAAGTGGGCTCAGCTACCTGATCCTGGTGAGCAGTACCCTACGT
BoESP3          TGCTGGGAAATGGGTTCAGCTACCAGATCCTGGAGTGCAGTTTGAGAAGT

BoESP1          TCGAGAGAAGAGGAGGAGCTGGATTCGTTGTGGTGCAAGGAAAGATTTGG
BoESP2          TCGAGAGAAGAGGAGGAGCTGGATTCATTGTGGTGCAAGGGAAGATTTGG
BoESP3          TCGAGAAAAGAGGTGGAGCTGGATTCGCTGTGGTGCAAGGAAAGATTTGG

BoESP1          GTGGTTTACGGGTTTGCAACTTCTCCTGATCCTAATGGAAAAAATGACTA
BoESP2          GTGGTTTACGGGTTTGCAACTTCACCTGATCCTAATGGAAAAAACGATTA
BoESP3          GTGATTTACGGGTTTGCGACTTCGCCTGATCCTAATGGGATGAACGACTA

BoESP1          TGAGTCCGACCAAGTGCAGTTTATGACCCGGCTACTCAAAAATGGACCG
BoESP2          TGAGTCTGATCTTGTGCACTACTTTGATCCTGCTACTCAAAAGTGGACCG
BoESP3          CGAGTCTGATCTTGTTCACTACTTTAATCCCGCTACTCAAAAATGGACCG

BoESP1          AAGTGGAGACTAAAGGAGACAAACCTTCTGCGAGGAGCGTGTTTGGACAT
BoESP2          AAGTGGAGACTAAAGGAGAGAAACCTTCTCCAAGAAGCGTGTTTGCGCAT
BoESP3          AAGTGGAGACCAAAGGACAGAAACCTTCTGCGAGGAGCGTGTTTGCGCAT

BoESP1          GCGGTTGTGGGGAAATATATACTGATATTTGGAGGAGAGACCTGGCCGGA
```

```
BoESP2          GCGGCAGTAGGAAAATACATAATAATATTTGGAGGTGAGGTCGGGCCGGA
BoESP3          GCGGTAGTAGGAAAATATATACTAATATTTGGAGGTGAGACCTGGCCGGA

BoESP1          CCCAAAGGCACATTTGGGACCAGGGACGTTGTCTGATGAAGGGTTTGCTT
BoESP2          TCCAAACGGACATTATGGTCCCGGAACATTGACCAATGAGGGTTACGCGT
BoESP3          CCCAAGGGCACATTTGGGTCCCGGGACGTTGTCCAGTGAGGGTTTTGCGT

BoESP1          TGGACACTGAGACACTGGTGTGGGAGAGGTTTGGAGGAGGAGCTGAACCG
BoESP2          TGAACACCGAGAGTTTGGTGTGGGAGAAGTTTGGAGGAGGAGCTGAACCG
BoESP3          TGAACACTGAGACATTGGTGTGGGAGAAGTTTGGAGGAGGAAATGAACCG

BoESP1          GGTCAACTCGGTTGGCCCGGTTACACGACTGCCACCGTGTATGGAAAGAA
BoESP2          GGTGAACTCGGTTGGCCTGCCTACACGACTGCTACCGTCTATGGAAAGCA
BoESP3          GGGCAACTTGGTTGGCCCGCCTACACGGCTGCCACCATCTATGGAAAGAT

BoESP1          AGGTCTCCTTATGCATGGGGGAAAACGTCCGACCAACAACCGAACCGATG
BoESP2          AGGCCTCCTCATGCATGGAGGAAAACGTCCCACCAACAACCGTACCGATG
BoESP3          AGGCCTCCTCATGCATGGAGGAAAGCGTCCTACCAATAACCGTACCGATG

BoESP1          AGCTCTACTTCTACGCAGTTAATTCCGCGTAA
BoESP2          AGATGTACTTCTACGCGGTCCATTCCGCCTAA
BoESP3          AGATGTACTTCTACGCAGTCAATCGCGCCTAA
```
