# Supplementary material for: Identification and Characterization of Three Epithiospecifier Protein Isoforms in Brassica oleracea
Source: Front Plant Sci. 2019 Dec 19;10:1552. doi: 10.3389/fpls.2019.01552 (PMC6930892; doi:10.3389/fpls.2019.01552)

Figure S2: Glucosinolate (GLS) substrate-specific nitrile specifier protein (NSP) activity of recombinant BoESP1-3 as assessed by the hydrolysis of non-alkenyl GLS to release the corresponding nitriles 5-(methylsulfanyl)butanenitrile (4MTB-CN; A), 4-(methylsulfinyl)butanenitrile (3MSOP-CN; B), 5-(methylsulfinyl)pentanenitrile (4MSOB-CN; C), and 3-phenylpropionitrile (2PE-CN, D). Values represent mean ± standard deviation of three independent experiments, comprising of 2-3 technical replicates each (total n=3). Different capital letters indicate significant differences in means between the formation of the nitrile from one GLS by the different BoESP and a water control as tested by ANOVA and Tukey test at the $p \leq 0.05$ level.

A

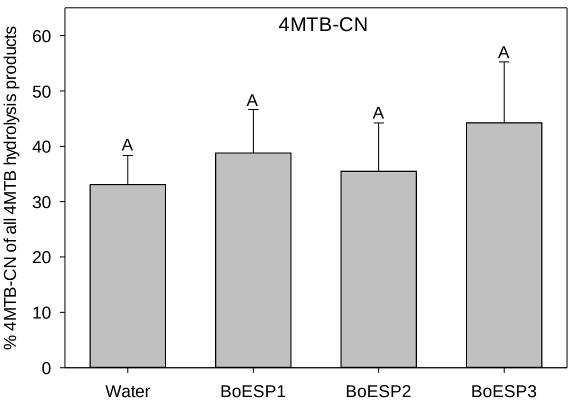

B

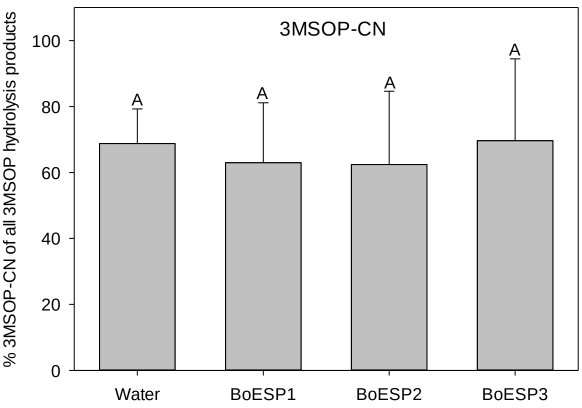

C

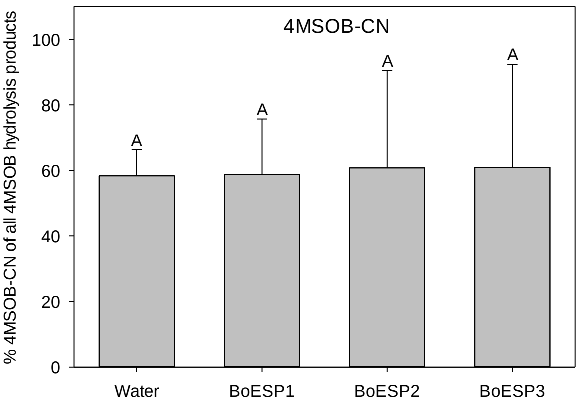

D

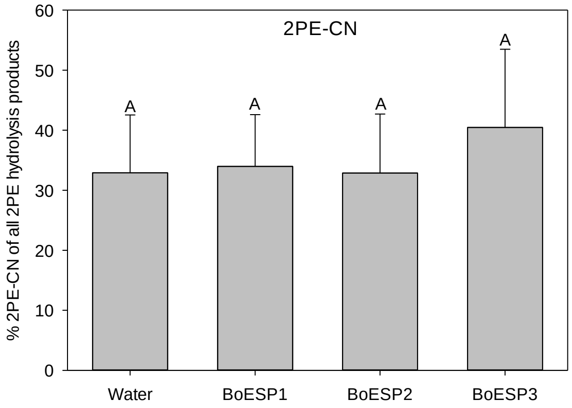

Supplement: Supplementary file 2 [file Image_2.pdf]
