# Supplementary material for: Identification and Characterization of Three Epithiospecifier Protein Isoforms in Brassica oleracea
Source: Front Plant Sci. 2019 Dec 19;10:1552. doi: 10.3389/fpls.2019.01552 (PMC6930892; doi:10.3389/fpls.2019.01552)
Supplement: Supplementary file 3 [file Image_3.pdf]

Figure S3:

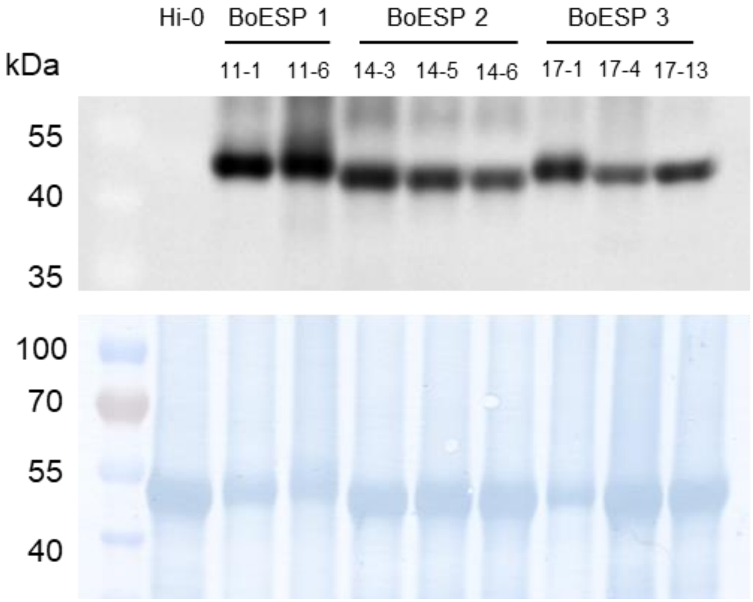

Figure S3: Characterization of transgenic Arabidopsis lines overexpressing BoESP proteins. Western blot analysis of 35S-BoESP-myc lines using an anti myc-antibody. Leaf material from 4-weeks old plants of the T2 generation was used for the analysis. Hi-0 represents the untransformed wild type control. Amido black stain of RuBisCo on the membrane served as loading control.
