# Supplementary material for: Identification and Characterization of Three Epithiospecifier Protein Isoforms in Brassica oleracea
Source: Front Plant Sci. 2019 Dec 19;10:1552. doi: 10.3389/fpls.2019.01552 (PMC6930892; doi:10.3389/fpls.2019.01552)

Figure S4: Formation of 8-(methylsulfanyl)octyl GSL (8MTO) hydrolysis products 8-(methylsulfanyl)octyl isothiocyanate (8MTO-ITC) and 9-(methylsulfanyl)nonanenitrile (8MTO-CN) in % relative to all 8MTP hydrolysis products in *Arabidopsis thaliana* Hi-0 control and BoESP1-3 transformant lines in shoot tissue (A) and root tissue (B). Values represent mean ± standard deviation of three independent experiments (n=3).

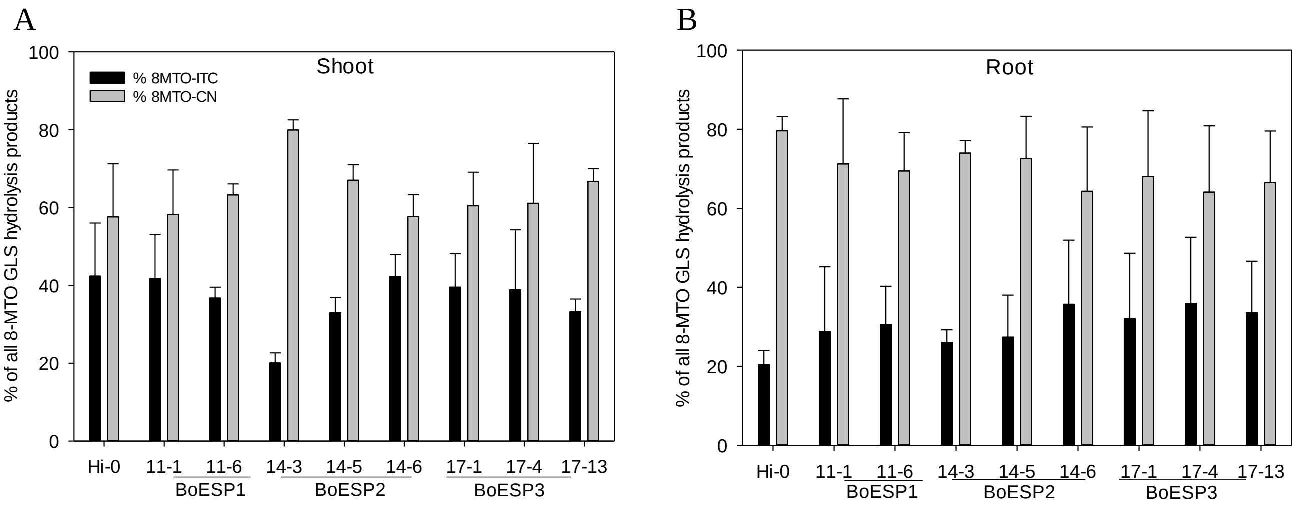

Supplement: Supplementary file 4 [file Image_4.pdf]
