# Supplementary material for: Identification and Characterization of Three Epithiospecifier Protein Isoforms in Brassica oleracea
Source: Front Plant Sci. 2019 Dec 19;10:1552. doi: 10.3389/fpls.2019.01552 (PMC6930892; doi:10.3389/fpls.2019.01552)
Supplement: Supplementary file 5 [file Table_1.docx]

| Gene name | Gene ID (NCBI) | Sequence | Annealing temperature (°C) | Product length (bp) |
| --- | --- | --- | --- | --- |
| *BoESP1* | LOC106296341 | 5‘-GCAGTTCCCAAGGTTCGAGA-3‘  5‘-TTCCATACACGGTGGCAGTC-3’ | 54 | 410 |
| *BoESP2* | LOC106306810 | 5‘-AGCAAAGGCGGGGTTATGAA-3‘  5‘-AACGCGTAACCCTCATTGGT-3‘ | 54 | 413 |
| *BoESP3* | LOC106325105 | 5‘-AAGGACCTGGACCAAGAAGC-3‘  5‘-ACAGGGGTTTTGTTCGTCCC-3‘ | 54 | 415 |
| *BoSAND1* | KF218596 | 5‘-CGCCAATCTGCCTACCAAGA-3‘  5‘-GGCCTCTGAACGTGTGGTAA-3‘ | 54 | 107 |
| *BoTUB6* | KF218597 | 5‘-CAATACCAAGACGCAACCGC-3‘  5‘-ACGCTAGTCTCAGCAGCATT-3‘ | 54 | 99 |
|  | LOC106297542 | 5‘-AGAGGGACCAGGACCAAGAA-3‘  5‘-GACCTCCCACTTCATCCAGC-3‘ | 54 | 323 |

Table S1: qPCR primer sequences, their annealing temperatures and the length of the resulting amplicon for *BoESP* transcript abundance.
