# Supplementary material for: Identification and Characterization of Three Epithiospecifier Protein Isoforms in Brassica oleracea
Source: Front Plant Sci. 2019 Dec 19;10:1552. doi: 10.3389/fpls.2019.01552 (PMC6930892; doi:10.3389/fpls.2019.01552)
Supplement: Supplementary file 6 [file Table_2.docx]

Table S2: Primers used for plasmid construction.

| Primer |  | Sequenz (5’-3’) | Description |
| --- | --- | --- | --- |
| BoESP1 | Fw | CACCATGGCTCCGAGTGTGCAAGG | Primer for plant expression construct |
|  | Rev | CGCGGAATTAACTGCGTAG |  |
| BoESP2 | Fw | CACCATGGCTCCCACATTGCAAGG | Primer for plant expression construct |
|  | Rev | GGCGGAATGGACCGCGTAG |  |
| BoESP3 | Fw | CACCATGGCTCCGACTTTGCAAGG | Primer for plant expression construct |
|  | Rev | GGCGCGATTGACTGCGTAG |  |
| BoESP1 | Fw | CACCATGGCTCCGAGTGTGCAAGG | Primer for *E. coli* expression construct |
|  | Rev | TTACGCGGAATTAACTGCGTAG |  |
| BoESP2 | Fw | CACCATGGCTCCCACATTGCAAGG | Primer for *E. coli* expression construct |
|  | Rev | TTAGGCGGAATGGACCGCGTAG |  |
| BoESP3 | Fw | CACCATGGCTCCGACTTTGCAAGG | Primer for *E. coli* expression construct |
|  | Rev | TTAGGCGCGATTGACTGCGTAG |  |
